# *Perk* Ablation Ameliorates Myelination in S63del-Charcot–Marie–Tooth 1B Neuropathy

**DOI:** 10.1177/1759091416642351

**Published:** 2016-04-14

**Authors:** Nicolò Musner, Mariapaola Sidoli, Desireè Zambroni, Ubaldo Del Carro, Daniela Ungaro, Maurizio D’Antonio, Maria L. Feltri, Lawrence Wrabetz

**Affiliations:** 1 Hunter James Kelly Research Institute, University at Buffalo, NY, USA; 2 Department of Biochemistry, Jacobs School of Medicine and Biomedical Sciences, University at Buffalo, NY, USA; 3 Division of Neuroscience, San Raffaele Scientific Institute, DIBIT, Milan, Italy; 4 Division of Genetics and Cell Biology, San Raffaele Scientific Institute, DIBIT, Milan, Italy; 5 Department of Neurology, Jacobs School of Medicine and Biomedical Sciences, University at Buffalo, NY, USA

**Keywords:** Charcot–Marie–Tooth, myelin, PERK, proteostasis, Schwann cells, unfolded protein response

## Abstract

In peripheral nerves, P0 glycoprotein accounts for more than 20% of myelin protein content. P0 is synthesized by Schwann cells, processed in the endoplasmic reticulum (ER) and enters the secretory pathway. However, the mutant P0 with S63 deleted (P0S63del) accumulates in the ER lumen and induces a demyelinating neuropathy in Charcot–Marie–Tooth disease type 1B (CMT1B)–S63del mice. Accumulation of P0S63del in the ER triggers a persistent unfolded protein response. Protein kinase RNA-like endoplasmic reticulum kinase (PERK) is an ER stress sensor that phosphorylates eukaryotic initiation factor 2 alpha (eIF2alpha) in order to attenuate protein synthesis. We have shown that increasing phosphophorylated-eIF2alpha (P-eIF2alpha) is a potent therapeutic strategy, improving myelination and motor function in S63del mice. Here, we explore the converse experiment: *Perk* haploinsufficiency reduces P-eIF2alpha in S63del nerves as expected, but surprisingly, ameliorates, rather than worsens S63del neuropathy. Motor performance and myelin abnormalities improved in S63del//*Perk*+/− compared with S63del mice. These data suggest that mechanisms other than protein translation might be involved in CMT1B/S63del neuropathy. In addition, *Perk* deficiency in other cells may contribute to demyelination in a non–Schwann-cell autonomous manner.

## Introduction

The peripheral nervous system (PNS) consists mainly of neurons and Schwann cells. Schwann cells produce myelin, which is a membranous structure composed by lipids and proteins that allows fast nerve conduction. The myelin protein zero gene (P0, *MPZ*) encodes the major protein constituent of myelin. Mutations affecting *MPZ* cause a wide array of hereditary neuropathies. In particular, deletion of serine 63 from P0 (P0S63del) causes Charcot–Marie–Tooth disease type 1B (CMT1B) neuropathy characterized by reduced motor capacity, defective nerve conduction, and demyelination (Kulkens et al., 1993; Miller et al., 2012).

Analysis of transgenic mice expressing this mutation revealed that P0S63del fails to reach myelin and is retained in the endoplasmic reticulum (ER) of Schwann cells (Wrabetz et al., 2006; Pennuto et al., 2008). The accumulation of P0S63del triggers the unfolded protein response (UPR), a set of signals aimed to restore cellular homeostasis (Schroder and Kaufman, 2005). Schwann cells and neurons are examples of factory cells; both face a high demand for protein synthesis and folding, rendering them particularly vulnerable to ER stress (D’Antonio et al., 2009; Gow and Wrabetz, 2009; Matus et al., 2011). If balance is not restored, terminal-phase UPR effectors may provoke cell death or dedifferentiation (Tabas and Ron, 2011; D’Antonio et al., 2013).

In mammals, there are three UPR transducers: Protein kinase RNA-like endoplasmic reticulum kinase (PERK), activating transcription factor 6 (ATF6), and inositol-requiring enzyme 1 (IRE1). IRE1 activates, through spliced X-box binding protein 1 (Xbp1s) transcription factor, the expression of genes involved in ER-associated degradation (ERAD; Friedlander et al., 2000; Travers et al., 2000). ATF6 promotes folding capacity by increasing the levels of ER-resident chaperones (Haze et al., 1999; Yoshida et al., 2000; Yoshida et al., 2001; Shen et al., 2002; Yamamoto et al., 2007). PERK represses translation of most messenger RNAs (mRNAs) by phosphorylating eukaryotic initiation factor 2 alpha (eIF2alpha), hence reducing the load of proteins in the ER lumen. Phosphorylated-eukaryotic initiation factor 2 alpha (P-eIF2alpha) protects and promotes survival in factory cells. In fact, the ability to cope with persistent ER stress is significantly decreased in *Perk*-deficient cells (Harding et al., 2001b; P. Zhang et al., 2002a). P-eIF2alpha simultaneously increases the translation of activating transcripton factor 4 (ATF4) that upregulates CCAAT/enhancer-binding protein homologous gene (*Chop*), an effector of cell death or dedifferentiation (Zinszner et al., 1998; Harding et al., 2000a; Tsang et al., 2007; Pennuto et al., 2008; D’Antonio et al., 2013). In S63del mice, genetic ablation of *Chop* rescued motor function and reduced demyelination (Pennuto et al., 2008). CHOP aggravates S63del neuropathy by upregulating growth arrest and DNA damage-inducible protein 34 gene (*Gadd34)*, encoding a regulatory subunit of protein phosphatase 1 (PP1) holophosphatase that reactivates protein translation via dephosphorylation of eIF2alpha.

We previously demonstrated that Gadd34 inactivation rescued S63del neuropathy by augmenting the level of P-eIF2alpha and, therefore, reducing protein synthesis and overload in the ER, and alleviating the UPR (D’Antonio et al., 2013). Thus, we reasoned that reduced phosphorylation of eIF2alpha would exacerbate S63del neuropathy. Nonetheless, we show here that *Perk* haploinsufficiency surprisingly ameliorates S63del myelin defects *in vivo* and *in vitro*, even if nerve levels of P-eIF2alpha were decreased. Moreover, *Perk* ablation improved motor capacity in S63del mice indicating that PERK is detrimental in CMT1B neuropathy. Our data indicate that improved S63del neuropathy is not always coupled to increased P-eIF2alpha levels in nerve.

## Materials and Methods

### Animals

All experiments involving animals were performed in accord with experimental protocols approved by the San Raffaele Scientific Institute Animal Care and Use Committee. S63del-L and S63del-H (hereafter, S63del and S63del-H) transgenic mice (129.4 and 129.1 lines, 60% and 210% overexpression of P0S63del, respectively; Wrabetz et al., 2006), P0-overexpressing (P0OE) mice (80.4 line; Wrabetz et al., 2000), and *Perk*-null mice (Harding et al., 2000b) have been described previously. P0S63del and P0OE were maintained on the FVB/N background, whereas *Perk*+/− mice, initially on the 129S6 background, were backcrossed for at least six generations onto the FVB/N background before initiation of experiments. S63del mice were crossed with *Perk*+/− mice in order to obtain wildtype (WT), S63del, *Perk*+/−, and S63del//*Perk*+/− genotypes. P0S63del transgene was detected in FVB background mice through Tg80 PCR, with the following primer sequences: P0 sense (5′-GAGATGCCATTTCGGTGAGT-3′) and P0 antisense (5′-GCTATTTGCCCTTCTCAGTC-3′). Following digestion with BamH1 restriction endonucleases, two bands are recognized for the positive pups; 310 bp (cut) + 400 bp. To detect the *Perk* KO allele, the following primers were used; PGK.255R (5′-GCTACCGGTGGATGTGGAATGTG-3′), PERK.i6AS (5′-CGGAGACAGTACAAGCGCAGATGA-3′), and mPERK1730S (5′-AAGGACCCTATCCTCCTGCTGCAC-3′). Expected bands were 230 bp (wt), 300 bp (null allele; Harding et al., 2000b). In all experiments, littermates were used as controls.

### PERK Immunoprecipitation

Where indicated, WT mice were injected intraperitoneally with 1 µg of tunicamycin in 150 mM dextrose/g body weight. Mice were killed by CO_2_ inhalation after 48 hr. Fragments of livers from WT or injected animals and a total number of 40 to 50 sciatic nerves from each of WT, S63del, S63del-H, and P0-OE mice at postnatal day 28 (P28) were harvested and snap-frozen in liquid nitrogen. Tissues were pulverized in a stainless steel mortar at −80℃, and the powder was homogenized with a motorized Teflon pestle with 1 ml of SDS-free buffer (1% Triton X100, 150 mM NaCl, 20 mM Hepes pH 7.5, 10% glycerol, 1 mM EDTA) containing phosphatase (1X, Phostop, Roche) and protease inhibitors (PIC, Sigma). The homogenate was centrifuged twice at 14,000 rpm at 4℃, and protein concentration was assessed by the BCA method (Pierce). An equal amount of lysate (2 mg for nerves and 4 mg for liver) was diluted to a final volume of 700 µl for each sample. For nerves, 20 µl were reserved for Western analysis to normalize the amount of protein. Saturation of endogenous immunoglobulins and aspecific binding were prevented by adding 50 µl of protein A beads (GE-Healthcare) and 1 µl of a nonspecific rabbit antibody to lysates and incubating for 1 hr at 4℃. Lysates were centrifuged and beads discarded. Rabbit polyclonal sera (2 µl) against PERK (or subsequently against general control nonderepressible 2 (GCN2), heme-regulated eIF2 alpha kinase (HRI), or protein kinase RNA (PKR) were added together with 50 µl of protein A beads to each sample and left rotating overnight at 4℃. After centrifugation at 14,000 rpm for 10 min, the PERK-containing beads were washed and the excess of washing solution was eliminated with a 27 G needle. Laemmli buffer was added (15–20 µl), and beads were boiled for 5 min and loaded onto a 6% to 7% SDS-PAGE gel for Western analysis.

### Western Analysis

Sciatic nerves from transgenic and WT animals were dissected and snap-frozen in liquid nitrogen. Proteins were extracted in 100 µl of SDS Lysis Buffer (25 mM Tris-HCl, pH 7.4, 95 mM NaCl, 10 mM EDTA, 2% SDS) with phosphatase (final 1X, Phostop, Roche) and protease inhibitors (final 1X, PIC, Sigma). Protein content was assessed by the BCA method (Pierce) following manufacturer’s instructions. Total proteins (10–40 µg) were resolved by 6–12% SDS-PAGE under denaturing conditions as previously described (Wrabetz et al., 2000). Mouse monoclonal antibodies recognized beta-tubulin (1:2,000; Sigma–Aldrich), eIF2alpha (L57A5, Cell Signaling, 1:1,000), and calnexin (1:500). Rabbit monoclonal antibodies recognized P-eIF2alpha (119A11, Cell Signaling, 1:500). Rabbit polyclonal antibodies recognized binding immunoglobulin protein (BiP; Novus Biologicals, 1:1,000), Gadd34 (10449-1-AP, Proteintech, 1:500) ATF4 (sc-200, Santa Cruz, 1:500), ATF6 (1:100), PKR (D-20, Santa Cruz, 1:500), P-Thr446-PKR (ab32036, Abcam, 1:500), and HRI (07-728, Millipore, 1:1,000). Rabbit poly-sera against PERK (1:1,000), GCN2 (1:1,000), and P-Thr898-GCN2 (1:500) were the generous gift of David Ron (Cambridge, UK). Suitable HRP-conjugated secondary antibodies (1:2,000–1:5,000) were visualized by ECL or ECL Plus (Pierce) in autoradiography (GE-Healthcare). Alternatively, rabbit or mouse 800CW/600CW IRDye conjugated antibodies (LI-COR; 1:5000) were revealed with the Odyssey 2.0 scanner (LI-COR).

### Behavioral, Electrophysiological, and Morphological Analyses

Motor capacity was assessed in 4-month-old mice by rotarod analysis, as described previously (Wrabetz et al., 2006) testing 16 to 23 animals/genotype. Electrophysiology was performed as previously described (Wrabetz et al., 2006), on five mice/genotype at 4 months of age (both sciatic nerves). An equal number of male and female animals were used to perform behavioral and electrophysiological analyses. The number of onion bulbs and demyelinated fibers were counted blind to genotype in toluidine blue–stained semi-thin sections in sciatic nerves of 12-month-old animals. A total of 42 to 64 fields were captured from three to four animals/genotype using a 100× objective. The measure of g-ratio was obtained as previously described using Qwin3 semi-automated system (Leica Microsystems; Pennuto et al., 2008). A total of 2,000 to 3,000 myelinated fibers obtained from four microscopic fields/nerve from three animals/genotype were analyzed.

### In-Vivo Internodal Length Evaluation

Femoral quadriceps nerves at P10 or P20 were fixed for 1 hr at room temperature in a 1% osmium-tetroxide/0.1 M phosphate buffer (pH 7.4) solution. Nerves were then washed twice and left at 50℃ for three overnight periods in ascending 60%, 80%, or 100% glycerol/0.1 M phosphate buffer (pH7.4) solutions. Single nerve fibers were teased from nerves with G27 syringe needles under a microscope, and brightfield images of at least 111 to 182 internodes (three animals/genotype) were measured with ImageJ software.

### Immunohistochemistry

Brain, cerebellum, and muscle were dissected from P28 mice and fixed in 10% formalin buffer overnight at room temperature. The sections were embedded in paraffin, and 4 -µm sections were cut and dried at 60℃ for 1 hr. Slides were cooled to room temperature, deparaffinized in three changes of xylene, and rehydrated using graded alcohols. Finally, the slides were stained with hematoxylin and eosin, rinsed with water, and coverslipped.

Fresh frozen P28 sciatic nerve sections (8–10 µm) were fixed with 4% paraformaldehyde (PFA) for 15 min and permeabilized with −20℃ cold methanol for 5 min. Blocking was performed for 30 min in undiluted goat serum followed by a 45-min incubation with bis(trimethylsilyl)acetamide (BSA) 1%–Triton 0.2% in phosphate-buffered saline (PBS). Rabbit anti-CHOP (1:200, gift of Alex Gow) and rat anti-myelin basic protein (MBP; 1:4, gift of Judith Grinspan) were incubated overnight in BSA 1%–Triton 0.2% in PBS. Suitable secondary antibodies (1:100–300) were then incubated for 1 hr. Sections were then washed, exposed to 4′,6-diamidino-2-phenylindole (1:10,000) and mounted in VectaShield medium (Vector Labs). To assess the nuclear localization of CHOP, confocal images were taken using Leica confocal SP2 microscope with the 60× objective. Staining of myelinated cultures was performed at 12 days after ascorbic acid induction. Dorsal root ganglia (DRG) neurons were fixed with ice-cold 4% PFA for 15 min and permeabilized for 5 min with −20℃ cold methanol. Coverslips were moved to a wet chamber and blocked with 10% donkey serum in PBS for 1 hr. Rat antibody against MBP (1:4, Judith Grinspan) and rabbit antibody against NF-H (1:200, EMD Millipore) were incubated overnight at 4℃ in BSA 1%–Triton 0.2%. Anti-rabbit FITC-conjuated (1:200, Jackson Immuno Research Labs) and DyLight 549-conjugated anti-rat antibodies (1:800) were incubated for 1 hr at room temperature. Coverslips were then exposed to DAPI and mounted as before. Only the areas where neurofilament staining (axons) was present were considered for counting. Coverslips were visualized on a DM5000B fluorescence microscope (Leica), images captured with a Leica DFC480 digital color camera and processed with Adobe Photoshop CS4. The internodal length was measured with ImageJ software.

### Myelinating DRG Explant Cultures and Cocultures

DRG from E13.5 WT, *Perk*+/−, *Perk*−/−, S63del, S63del//*Perk*+/− and S63del//*Perk*+/− embryos were cultured as previously described (Taveggia et al., 2005). After 6 days in culture, myelination was induced with 50 µg/ml ascorbic acid (Sigma–Aldrich) and the number of myelinated segments was assessed after 12 days upon addition of ascorbic acid. Pure DRG neuronal cultures were obtained as previously described (Taveggia et al., 2005) using three 2-day cycles of normal or FudR–supplemented media. Rat Schwann cells (2 × 10^5^) were seeded on pure neuronal cultures and left to recover for 6 days. Afterward, Schwann cells were induced to myelinate with 50 µg/ml ascorbic acid in culture media and blocked at 21 days after seeding for assessment. Parallel, non-seeded neuronal cultures certified absence of mouse Schwann cells. The number and length of MBP-positive myelinated internodes (see Immunohistochemistry section) was counted from 8 to 10 fields per DRG and from 8 to 12 DRG per embryo. Three to nine embryos per genotype were dissected in four separate experiments.

### TaqMan® Quantitative PCR Analysis

Total RNA from mouse P28 sciatic nerves was extracted using TRIzol (Roche), as previously described (Pennuto et al., 2008). Synthesis of cDNA was performed starting from 2 µg of total RNA using a final concentration of 50 µg/µl oligo dT (Promega), 5 µg/µl random hexamers (Promega), 2.5 mM dNTPs (Invitrogen), 30 U RNAsin (Promega), 0.02 M DDT, and 200 U Superscript-II reverse transcriptase (Invitrogen). The reaction mixture was incubated for 1 hr at 42℃. A 1:50 dilution of cDNA was prepared and 4 µl was used to perform the quantitative PCR. The ABI PRISM 7700 sequence detection system (Applied Biosystems Instruments) was used for the amplification according to the manufacturer’s instructions (TaqMan®; Applied Biosystems). The relative standard curve method was applied using WT mice as reference. Normalization was performed using 18 S rRNA. Target and reference amplifications were performed in separate wells with TaqMan® Assays on Demand (Life Technologies): 18 S, Hs99999901_s1; Chop, Mm00492097_m1; Myd116/Gadd34, Mm00435119_m1; Atf4, Mm00515324_m1; Xbp-1 s, Mm03464496_m1.

### Statistical Analysis

In every experiment, at least three biological replicates were analyzed. To determine the significance between WT and S63del or S63del and S63del//*Perk*+/− Student’s *t* test or one-way analysis of variance (ANOVA) were used. *p* ≤ .05 was considered statistically significant. Graphical data are represented as mean ± *SEM*.

## Results

The UPR initiates when misfolded proteins accumulate in the ER (Ron and Walter, 2007). Cells induce translational attenuation as a fast and efficient strategy to prevent the accumulation of client proteins in the ER and cope with stress (Harding et al., 2001a). Complex eukaryotes achieve global attenuation of protein translation by activating the PERK P-eIF2alpha pathway (Harding et al., 2001a). Misfolded P0S63del accumulates in the ER of Schwann cells, eliciting a chronic UPR that causes CMT1B neuropathy (Wrabetz et al., 2006; Pennuto et al., 2008). Indeed, genetic and pharmacological inhibition of Gadd34 in S63del mice resulted in increased levels of P-eIF2alpha, lowered the rates of protein synthesis, and rescued the S63del phenotype (D’Antonio et al., 2013). To understand if, on the contrary, reduced eIF2alpha phosphorylation would worsen S63del neuropathy, we bred S63del mice with *Perk+*/− mice, where PERK-dependent phosphorylation of eIF2alpha is impaired (Harding et al., 2001b). Unfortunately, we could not study nerves from full *Perk* ablation because *Perk*−/− mice invariably died soon after birth in the FVB/NCrl background (Harding et al., 2001a).

### Perk is Necessary for eIF2alpha Phosphorylation in S63del Nerves

**Figure 1.**
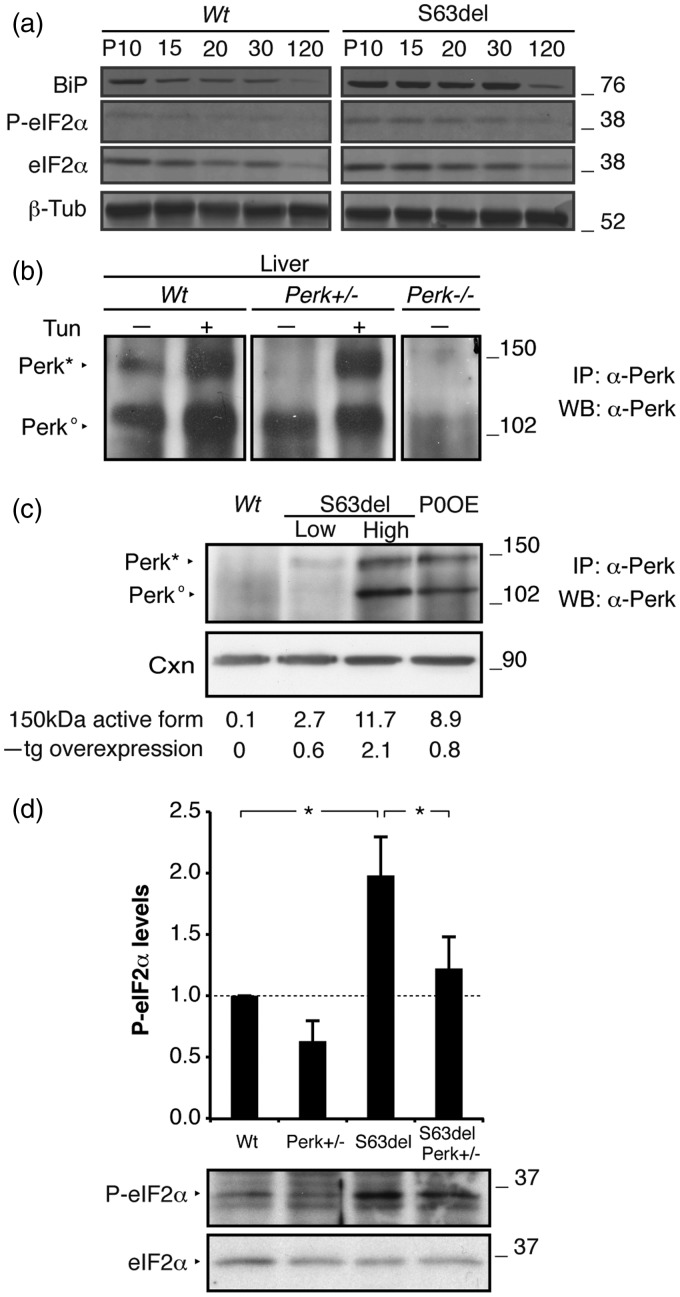
PERK is required for eIF2alpha phosphorylation in S63del nerves. (a) Western blot analysis on P10 to 4-month-old (P120) sciatic nerve lysates was performed for the ER stress marker BiP, P-eIF2alpha, and total eIF2alpha. B-Tubulin (B-Tub) provided the loading control. (b) Protein lysates from liver of WT and *Perk*+/− animals injected (+) or not (−) with tunicamycin were immunoprecipitated and blotted with an anti-PERK antibody. A lysate from a *Perk*−/− embryo provided the negative control. PERK*, active form. PERK^0^, inactive form. (c) Immunoprecipitation and western blot for PERK on P28 sciatic nerve extracts. S63del low and high are two different transgenic lines expressing the P0S63del transgene at different levels (60%–210% overexpression, respectively). Densitometric analysis of the active 150 kDa form shows a P0S63del dose-dependent activation of PERK. Calnexin (Cxn) was used as loading control. (d) Western blot analysis for P-eIF2alpha on P28 sciatic nerves; one representative experiment of six is shown. P-eIF2alpha levels were measured by densitometric analysis. Error bars, *SEM*; *p < .05; by Student’s *t* test. *n* = 6. Numbers represent relative molecular weights.

To test PERK activation in the CMT1B mouse model, we analyzed the levels of P-eIF2alpha in S63del nerves between postnatal day 10 (P10) and 4 months of age. The phosphorylation of eIF2alpha was high in S63del nerves compared with WT between P10 and P30 (Figure 1a). Similar to P-eIF2alpha, also the chaperone BiP, a marker of ER stress, was highly induced in S63del nerves between P10 and P30, suggesting that P10–P30, when P0S63del is highly synthesized causing a chronic UPR, was a good timeframe for directly testing PERK activation.

Immunoprecipitation and Western blot analysis allowed us to distinguish the active, phosphorylated form of PERK (PERK*), from the inactive counterpart (PERK^0^) in P30 sciatic nerves (Figure 1b–c). Liver extracts derived from tunicamycin-injected mice served as positive controls (Figure 1b). In accordance with P-eIF2alpha levels, also PERK activation (PERK*) increased in a dose-dependent manner in S63del and S63del-H nerves (60% and 210% overexpression of *Mpz*S63del, respectively; Wrabetz et al., 2006) as compared with WT at P30. Interestingly, also P0OE nerves (80% overexpression; Wrabetz et al., 2000), showed increased PERK activation (Figure 1c), although full activation of a canonical UPR has not been detected in P0OE nerves (Pennuto et al., 2008; D’Antonio et al., 2013). Furthermore, the activation of the other three eIF2alpha kinases, GCN2, HRI, and PKR (Harding et al., 1999; Harding et al., 2000a; F. Zhang et al., 2001; P. Zhang et al., 2002b), was not significant in S63del nerves (Supplementary Figure 1) suggesting that PERK is the main eIF2alpha kinase active in S63del-CMT1B nerves. Consistent with these observations*, Perk* partial loss of function in S63del mice (S63del//*Perk*+/−) was sufficient to reduce P-eIF2alpha by roughly 40% in S63del nerves at P28 (Figure 1d). We conclude that PERK is the main kinase involved in eIF2alpha phosphorylation *in vivo*, and that we can experimentally explore the consequences of reduced P-eIF2alpha levels in the context of the UPR in S63del nerves.

### Perk Haploinsufficiency Paradoxically Ameliorates S63del Motor Performance and Reduces Demyelination and Onion Bulbs

**Figure 2.**
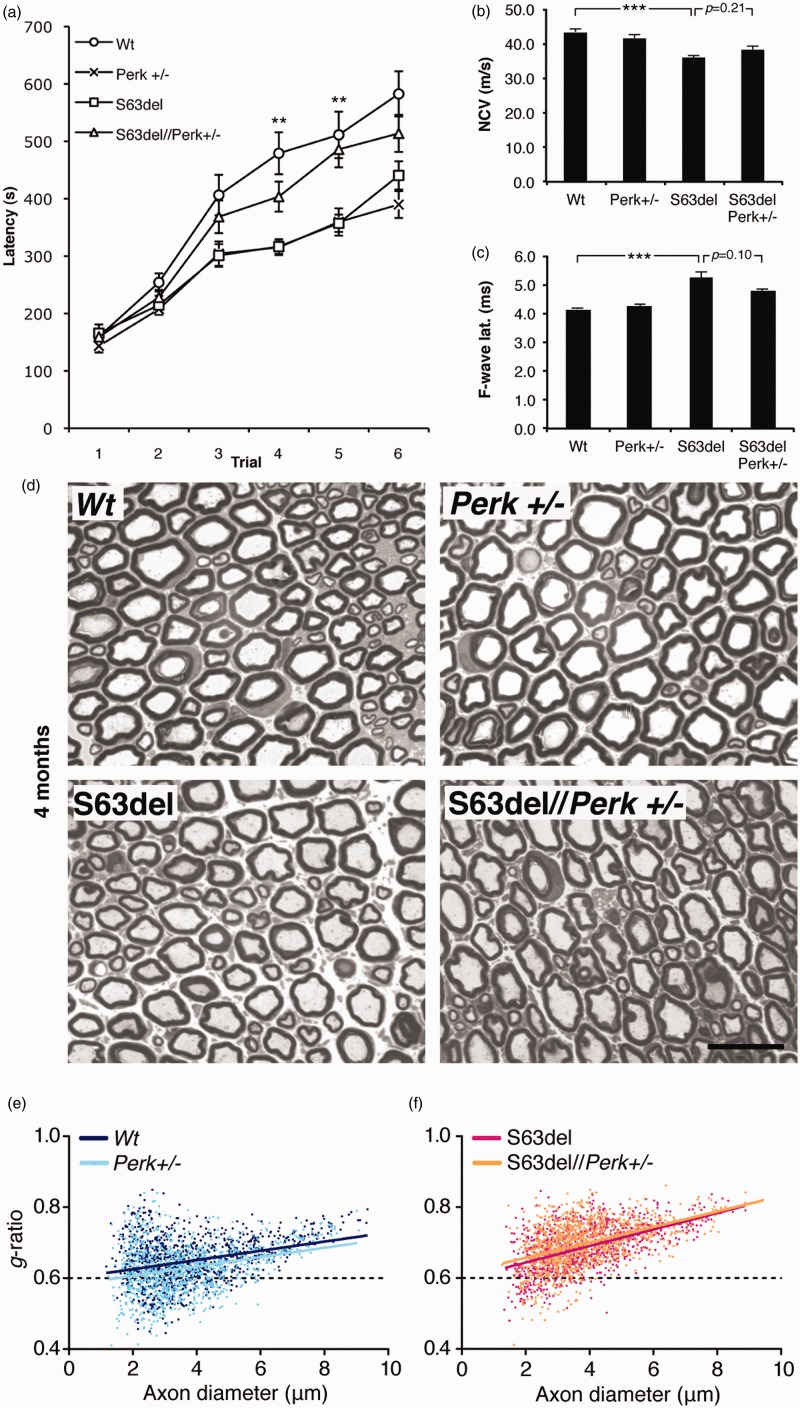
*Perk* ablation ameliorates motor function but not NCV and hypomyelination in S63del mice. (a) Rotarod analysis of motor function in WT, *Perk+/−*, S63del, and S63del//*Perk*+/− mice at 4 months. Error bars, *SEM*; **p < .01 for S63del//*Perk*+/− relative to S63del; *n* = 16 to 23 animals. (b) and (c) Electophysiological analysis showing NCV and F-wave latency (ms); Error bars, *SEM*; ***p < .001 by Student’s *t* test; *n* = 10 nerves from five animals. (d) Semi-thin sections stained with toluidine blue from 4-month-old WT, *Perk+/−*, S63del, and S63del//*Perk*+/− sciatic nerves. Bar, 20 microns. (e) and (f) Scatter plot of g-ratios as a function of axonal diameter measured in WT, *Perk+/−*, S63del, and S63del//*Perk*+/− mice at 4 months old (*n* = 3).

S63del mice display a progressive neuropathy characterized by impairment in motor capacity that can be detected by rotarod analysis around 4 months of age (Wrabetz et al., 2006; Pennuto et al., 2008; D’Antonio et al., 2013). The rotarod test of motor performance also successfully discriminated functional changes of S63del neuropathy in previous studies where the UPR was genetically perturbed (Pennuto et al., 2008; D’Antonio et al., 2013). Thus, to test whether *Perk* ablation would have a detrimental effect on S63del motor function, as expected from a resulting decrease in P-eIF2alpha, we performed rotarod analysis on WT, *Perk*+/−, S63del, and S63del//*Perk*+/− mice at 4 months of age. Surprisingly, S63del//*Perk*+/− animals showed a significant improvement in motor performance as compared with S63del mice (Figure 2a). Supporting the rotarod data, in S63del//*Perk*+/− mice F-wave latency showed a trend toward amelioration as compared with S63del (*p* = .10; Figure 2c), however, nerve conduction velocity (NCV) was not improved (Figure 2b).

Notably, *Perk*+/− mice showed reduced motor capacity, although NCV and F-wave latency were not significantly affected, suggesting a possible detrimental effect of *Perk* haploinsufficiency in tissues other than sciatic nerve that could affect the motor performance (Figure 2b–c). To verify this hypothesis, we performed morphological analysis of cerebellum, spinal cord, and gastrocnemius muscle (Supplementary Figures 2–4). Hematoxylin and eosin staining did not reveal gross abnormalities in the cerebellum and spinal cord suggesting that these tissues did not contribute to the reduced motor capacity of *Perk*+/− mice. Interestingly, *Perk*+/− mice displayed a trend toward a slightly increased number of centrally nucleated muscle fibers as compared with WT (Supplementary Figure 4a, inset a, black arrowheads), although a similar 1–2% of centrally nucleated fibers have been reported in WT mouse muscle (Sonnemann et al., 2006) suggesting that this slight increase is unlikely to explain significantly impaired rotarod performance.

Finally, metabolic diseases like diabetes mellitus are associated with neuropathy. *Perk*+/− mice have been reported to manifest glucose intolerance with only slightly elevated beta-islet cell death (Harding et al., 2001b). Accordingly, we found no obvious morphological difference in hematoxylin and eosin–stained pancreatic islets among WT, *Perk*+/−, S63del, and S63del//*Perk*+/− mice (data not shown).

S63del neuropathy is characterized by thinner myelin (hypomyelination; Pennuto et al., 2008; D’Antonio et al., 2013). We therefore investigated the effect of *Perk* loss of function in S63del nerves and quantified myelin thickness at 4 months. As expected, the g-ratio analysis revealed hypomyelination in S63del nerves compared with WT (0.69 ±  0.002 vs. 0.65 ±  0.002, *p* < .001; Figure 2e and f), but did not reveal a difference between S63del//*Perk*+/− and S63del nerves (Figure 2f). A very slight hypermyelination was instead detected in *Perk*+/− nerves when compared with WT (0.63 ±  0.002 vs. 0.65 ±  0.002, *p* < .001; Figure 2e).

**Figure 3.**
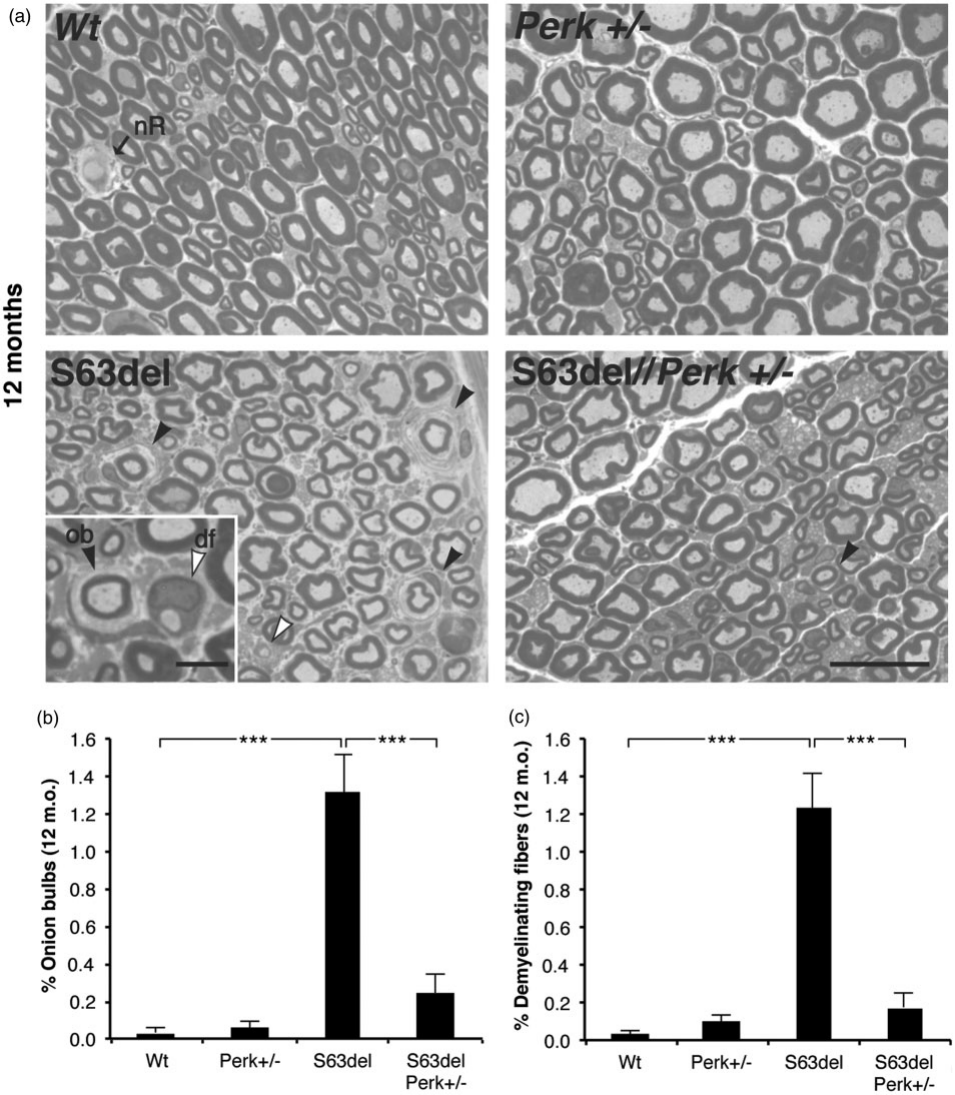
*Perk* ablation reduces demyelination and onion bulbs in S63del nerves. (a) Semi-thin sections stained with toluidine blue from 12-months-old WT, *Perk+/−*, S63del, and S63del//*Perk*+/− sciatic nerves. Arrows indicate nR; full arrowheads, ob; and empty arrowheads, df. Bar, 20 microns; inset bar 5 microns. (b) and (c) Quantification of ob and df. Error bars, *SEM*; ***p < .001, by Student’s *t* test; *n* = 42 to 64 fields derived from three to four animals per genotype. df = demylinated fibers; nR = nodes of Ranvier; ob = onion bulbs.

After 6 months of age, S63del nerves show progressive signs of demyelination and other pathological hallmarks, such as onion bulbs (Wrabetz et al., 2006). As previously reported, *Chop* ablation and the functional impairment of Gadd34 reduced the amount of demyelinated fibers and onion bulbs in S63del animals (Pennuto et al., 2008; D’Antonio et al., 2013). To further analyze the unexpected finding that *Perk* deficiency could rescue S63del neuropathy, we counted the number of demyelinating fibers and onion bulbs at 12 months, when they are more evident. Strikingly, both onion bulbs and demyelinated fibers were strongly diminished in S63del//*Perk*+/− sciatic nerves as compared to S63del (Figure 3). Only rare pathological findings were detected in *Perk*+/− and WT nerves. Consistent with this improvement, *Perk* deficiency in S63del produced a trend toward amelioration of the reduced myelin thickness observed in S63del compared with WT, as shown by g-ratio measurement in 12-month sciatic nerves (Supplementary Figure 5). Taken together, these data demonstrate that *Perk* haploinsufficiency does not worsen, but rather ameliorates S63del neuropathy.

### Amelioration of S63del Neuropathy does not Depend on Perk Deficiency in Neurons

Neither the worsening of *Perk*+/− motor performance, nor the amelioration of myelination in S63del//*Perk*+/− nerves was associated with obvious effects in the nervous system outside of peripheral nerve. Therefore, to investigate the effects further in nerve, we analyzed *Perk* ablation in nerve cells. We isolated myelinating DRG explants from E13.5 embryos. Importantly, since *Perk* null embryos are still viable at this stage, we could also test the full *Perk* ablation.

**Figure 4.**
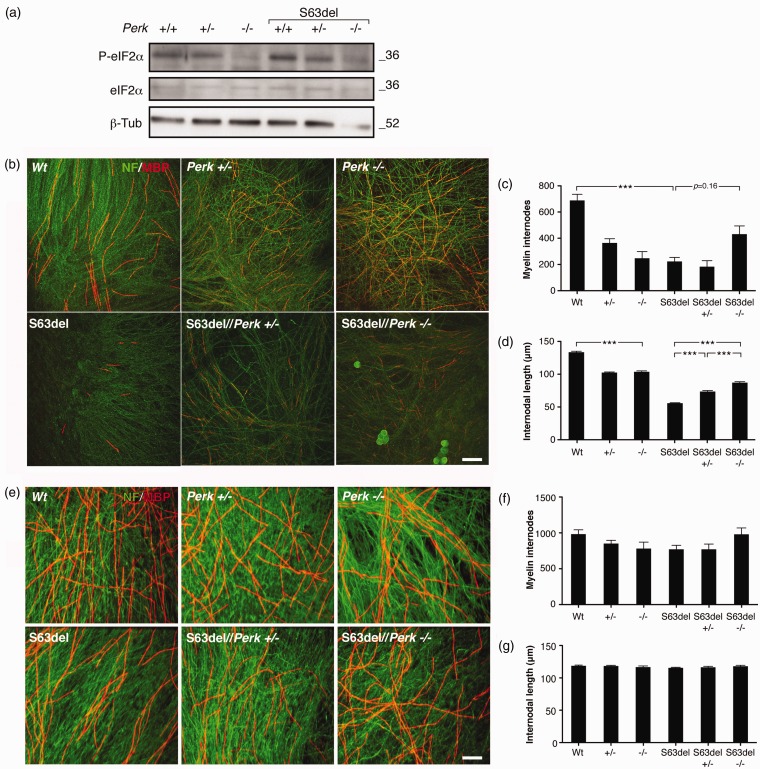
Ablation of *Perk* ameliorates S63del myelination in DRG explant cultures. (a) Western blot analysis of P-eIF2alpha levels on lysates of WT, *Perk+/−*, *Perk−/−*, S63del, S63del//*Perk*+/− and S63del//*Perk*+/− myelinating DRG explant cultures. Numbers represent relative molecular weights. (b) Confocal images of myelinating DRG explant cultures. Myelin internodes are stained for MBP (red), whereas neurites are stained for NF-H (green). Bar 50 microns. (c) and (d), quantification of the number of myelinated internodes and of the internodal length in DRG explant cultures. Error bars, *SEM*; ***p < .001; nested ANOVA considering intra- and inter-embryo variability; *n* = 3 to 9 embryos per genotype. (e) Confocal images showing purified WT, *Perk+/−*, *Perk−/−* DRG neurons seeded with rat Schwann cells. Myelin is visualized with MBP (red), and neurites with NF (green) Bar: 50 microns. (f) and (g) Quantification of the number of myelinated internodes and internodal length of DRG explant cultures. Error bars, *SEM*; *n* = 3 to 9 embryos per genotype.

Consistent with what we had observed *in vivo* in sciatic nerves, P-eIF2alpha levels were higher in S63del explants as compared with WT, and they decreased in a dose-dependent manner in *Perk* heterozygous and homozygous null explants (Figure 4a). As previously reported, in S63del explants, myelin segments are fewer than in WT explants (D’Antonio et al., 2013). Surprisingly, also *Perk*+/− and *Perk*−/− explants form less and shorter myelin internodes as compared with WT, whereas S63del//*Perk*+/− and S63del//*Perk*−/− explants have the tendency to form more and significantly longer internodes when compared with S63del (Figure 4b–d). The reduced number of myelinated segments in *Perk*+/− and *Perk*−/− as well as S63del DRG explants could not be explained by a diminished amount of Schwann cells or by a reduced number of axons because DAPI and neurofilament staining showed equal density of nuclei and of axons in all genotypes (data not shown).

To analyze whether this modulation of myelination was neuron cell-autonomous, we isolated mouse DRG explants from WT, *Perk*+/− and *Perk−/*− embryos, depleted them of endogenous Schwann cells and seeded them with WT rat Schwann cells. In this way, only DRG neurons are lacking *Perk,* as the rat Schwann cells are WT. To control for possible insertional effects of S63del transgene, we also processed analogously S63del, S63del//*Perk*+/−, and S63del//*Perk*+/− explants. The number of myelinated internodes and internodal length were unaffected in all of the explants, showing that neither the depletion of *Perk* in neurons nor S63del transgene insertion had an effect on Schwann-cell myelination (Figure 4e–g). These data suggest that *Perk* ablation has an important role in influencing myelination in S63del DRG explants and that Schwann cells might be primarily responsible for this effect.

**Figure 5.**
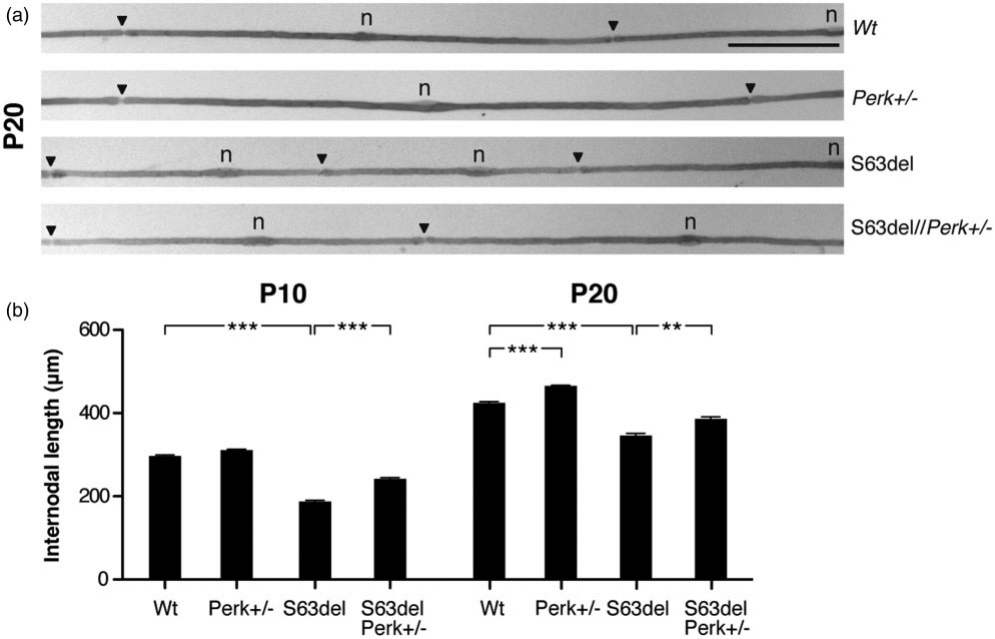
*Perk* ablation increases internodal length *in vivo*. (a) Osmicated teased fibers from WT, *Perk+/−*, S63del, and S63del//*Perk*+/− P20 femoral quadriceps nerves. Arrowheads, nodes of Ranvier; *n*, Schwann cell nuclei. Bar: 100 microns. (b) Quantification of internodal lengths at P10, P20, and P30. Error bars, *SEM*; **p < .01; ***p < .001; ns, not significant, one-way ANOVA; *n* = 111 to 182 internodes from at least three animals per genotype.

To confirm the *ex vivo* observations *in vivo*, we measured the internodal length of teased fibers from WT, *Perk+/*−, S63del, and S63del//*Perk*+/− sciatic nerves. Internodal length increases strongly between P5 and P20 and reaches its maximum around P30, when it stabilizes (Court et al., 2004). In agreement with what we found in the myelinating DRG explant cultures, S63del internodes are shorter than those in the WT. In addition, S63del//*Perk*+/− teased fibers show longer internodes than S63del between P10 and P20 (Figure 5a and b). To further support the *in vivo* data on S63del//*Perk*+/− myelination, we quantified the protein expression of MAG, MBP, and PMP22 myelin proteins in 4-month nerves. Western blot analysis showed a reduction in MBP levels in S63del nerves relative to WT, whereas levels of myelin-related proteins remained similar between S63del and S63del//*Perk*+/− (Supplementary Figure 6). As demyelination and onion bulbs occur in a small subset of fibers in S63del nerves, it is possible that these morphological features do not produce discernible effects in western analysis for myelin proteins. Nonetheless, our observations reinforce that, both *in vivo* and *ex vivo,* S63del myelination is ameliorated by *Perk* genetic ablation.

### Amelioration of the S63del//*Perk*+/− Phenotype is not Dependent on Chop Loss-of-Function

**Figure 6.**
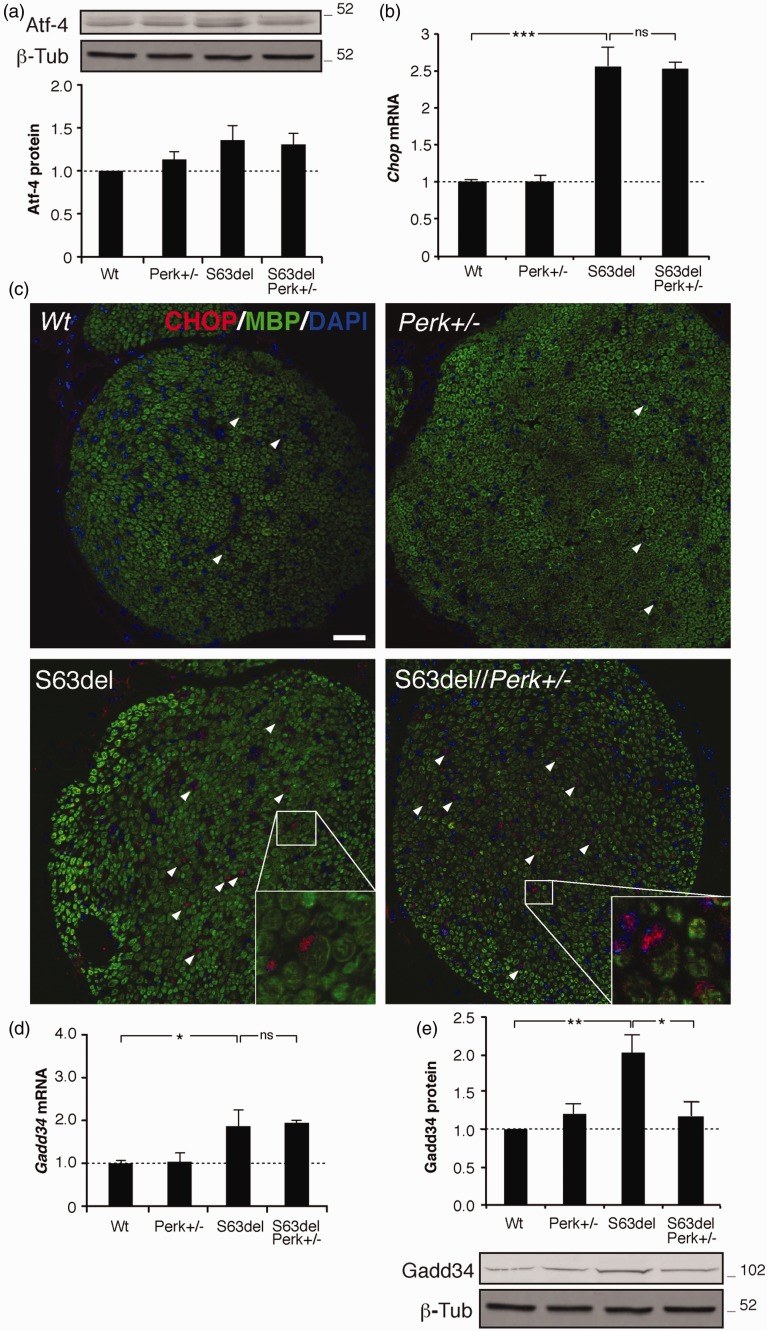
CHOP function is not altered in S63del//*Perk*+/− mice. (a) WB analysis on P28 sciatic nerves for ATF4; protein levels were quantified by densitometric analysis. One representative blot of three independent experiments is shown. Error bars, *SEM*; *n* = 3. Loading control, beta tubulin. (b) Quantitative RT-PCR for *Chop* mRNA from WT, *Perk+/−*, S63del, and S63del//*Perk*+/− sciatic nerves at P28. Error bars, *SEM*; ***p < .001, by Student’s *t* test; *n* = 3 animals for each genotype. (c) Confocal immunofluorescence for CHOP (red) on transverse sections of P28 sciatic nerves. MBP (green) identifies myelinating cells; nuclei are visualized with DAPI (blue). Arrowheads indicate CHOP-positive nuclei. A typical image of one out of three independent experiments on separate animals is shown. As controls, S63del-H (210% overexpression), and *Chop−/−* showed strongly positive and negative signals, respectively (not shown)*.* Bar: 50 microns. (d) Quantitative RT-PCR for *Gadd34* mRNA from WT, *Perk+/−*, S63del and S63del//*Perk*+/− sciatic nerves at P28. Error bars, *SEM*; *p < .05; Student’s *t* test; ns, nonsignificant; *n* = 3 independent replicates. (e) WB analysis on P28 sciatic nerves was performed for Gadd34; protein levels were quantified by densitometric analysis. β-Tub was used as loading control. One representative blot of seven independent experiments is shown. Error bars, *SEM*; *n* = 7. Numbers represent relative molecular weights. DAPI = 4′,6-diamidino-2-phenylindole; MBP = myelin basic.

The amelioration that we observed in S63del//*Perk*+/− mice closely resembles that detected in S63del//*Perk*+/− mice: S63del//*Perk*+/− animals also showed rescued motor capacity, improved F-wave latency and a reduction in demyelinated fibers (Pennuto et al., 2008). The transcription factor ATF4 lies between P-eIF2alpha and CHOP in the PERK signaling pathway. As P-eIF2alpha levels are reduced in S63del//*Perk*+/− nerves, stress-enhanced translation of ATF4 may be limited, reducing upregulation of *Chop* and its detrimental effect on myelination. To test this hypothesis, we measured ATF4 protein levels in sciatic nerves of P28 animals. Surprisingly, ATF4 is not decreased in S63del//*Perk*+/− nerves as compared with S63del (Figure 6a). Accordingly, *Chop* mRNA is still highly induced in S63del//*Perk*+/− nerves, suggesting that *Perk* haploinsufficiency does not have a strong influence on *Chop* upregulation.

CHOP must translocate into the nucleus in order to act as a transcription factor (Ron and Habener, 1992). To test whether *Perk* ablation could somehow impede Chop nuclear translocation, we performed CHOP immunostaining on sciatic nerve sections from *WT*, *Perk+/*−, S63del, and S63del//*Perk*+/− mice (Figure 6c). In S63del//*Perk*+/− nerves, CHOP localizes in Schwann cell nuclei similarly to S63del (Figure 6c, insets). Finally, to verify whether Chop is still able to up-regulate its target genes, we tested the expression of *Gadd34*, a target gene of Chop. *Gadd34* mRNA levels are elevated in S63del nerves (D’Antonio et al., 2013), and its levels remain elevated comparably in both S63del and S63del//*Perk*+/− nerves (Figure 6d). In addition, *Doc1, 4*, and *6*, other CHOP target genes, were not decreased (data not shown). These data suggest that CHOP remains equally functional in S63del and S63del//*Perk*+/− nerves. Gadd34 protein levels were instead significantly decreased in S63del//*Perk*+/− mice as compared with S63del, consistent with the fact that Gadd34 levels are also known to be regulated post-transcriptionally (Figure 6e).

These results suggest that, although S63del//*Perk*+/− mice seem to phenocopy S63del//*Perk*+/− mice, the amelioration of neuropathy observed here is likely to be independent from *Chop*. Therefore, we considered other candidates for S63del//*Perk*+/− rescue, for example, in the other UPR pathways.

### Activation of Other UPR Sensors is Not Perturbed in Response to Perk+/− Haploinsufficiency

**Figure 7.**
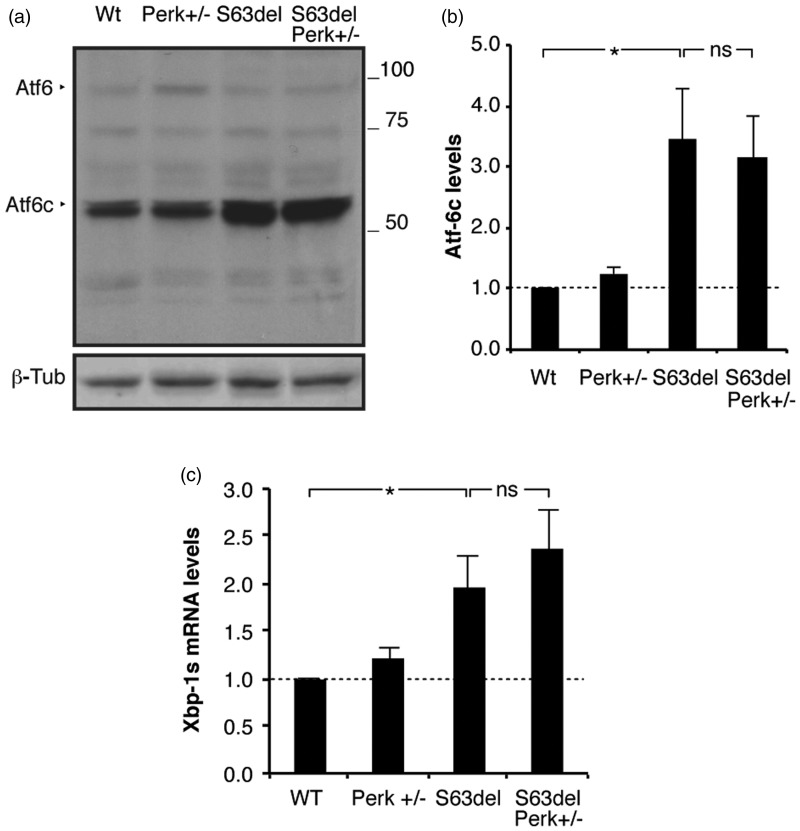
The ATF6 and IRE1 branches of the unfolded protein response are not affected by *Perk* ablation. (a) ATF6 cleavage analyzed by Western blot in WT*, Perk*+/*−*, S63del, and S63del//*Perk*+/− sciatic nerves at P28. One representative blot out of six independent experiments is shown. ATF6, uncleaved band; ATF6c, cleaved band. β-Tub provided the loading control. Numbers represent relative molecular weights. (b) ATF6c (active) band levels as measured by densitometric analysis. Error bars, *SEM*; *, *p* < .05; ns, non significant, by Student’s *t* test*; n* = 6. (c) Quantitative RT-PCR for XBP1s mRNA from WT, *Perk+/−*, S63del, and S63del//*Perk*+/− sciatic nerves at P28. Error bars, *SEM*; *n* = 6.

When challenged with ER stressors, *Perk*-null cells activate IRE1 more quickly and deactivate it more slowly compared with control cells, suggesting a compensatory overactivation of parallel UPR transducers in the absence of *Perk* (Harding et al., 2000b). Moreover, in cells with defective PERK signaling, increased activity of ATF6 and IRE1 stress pathways have been reported (Yamaguchi et al., 2008). We therefore hypothesized that *Perk* loss of function can ameliorate S63del myelination by augmenting folding capacity or ERAD, downstream of the other two UPR transducers. To test whether ATF6 or IRE1 pathways are regulated in response to *Perk* loss of function, we measured the levels of cleaved ATF6 and Xbp1 splicing. ATF6 does not respond to *Perk* heterozygosity, as cleaved ATF6 protein levels remain comparable in S63del and S63del//*Perk*+/− nerves (Figure 7a and b). Similarly, Xbp-1 splicing did not show overt alterations between S63del//*Perk*+/− nerves (Figure 7c). These data suggest that ATF6 and IRE1 branches of the UPR do not compensate for *Perk* haploinsufficiency and to improve the neuropathy.

## Discussion

Transgenic mice expressing P0S63del manifest a demyelinating neuropathy that mirrors CMT1B disease in humans (Wrabetz et al., 2006). In S63del peripheral nerves, P0 accumulates in the ER of Schwann cells, eliciting an UPR (Wrabetz et al., 2006). Genetic or pharmacological impairment of Gadd34 in S63del nerves, which should increase eIF2alpha phosphorylation, and reduce the synthesis of P0S63del, fully rescued this neuropathy phenotype (D’Antonio et al., 2013; Das et al., 2015). To further explore this relationship, we performed the converse experiment. Here, we report that crossing S63del mice with *Perk*-deficient mice reduces eIF2alpha phosphorylation in nerve by 40%, but surprisingly, ameliorates rather than worsens S63del motor function and its morphological hallmarks such as onion bulbs and demyelinated fibers. Moreover, also myelin internodal length increases significantly. These data suggest that the regulation of eIF2alpha phosphorylation might not be the only mechanism involved in the CMT1B neuropathy.

### PERK is the Main Kinase Phosphorylating eIF2alpha in Nerve

eIF2alpha phosphorylation can be enhanced by several stimuli, being the common target of the integrated stress response (Harding et al., 2003; Dever et al., 2007). At least four kinases phosphorylate eIF2alpha. PKR responds to viral dsDNA (F. Zhang et al., 2001); GCN2 and HRI respond to amino acid or heme deprivation (Harding et al., 1999; Harding et al., 2000a; P. Zhang et al., 2002b), whereas PERK reacts to ER accumulation of unfolded proteins (Harding et al., 2000b; P. Zhang et al., 2002a). Immunoprecipitation experiments show that PERK is the eIF2alpha kinase most abundant in S63del peripheral nerves and that its activation parallels the dosage of the S63del transgene (Figure 1c). Accordingly, eIF2alpha phosphorylation decreases in S63del//*Perk*+/− nerves and S63del//*Perk*+/− myelinating DRGs (Figure 4a). These results indicate that PERK is the main kinase involved in eIF2alpha phosphorylation in S63del neuropathy.

Interestingly, PERK is strongly active in P0OE nerves where P0WT is overexpressed (Figure 1c). We had previously reported that P0WT overexpression weakly, but significantly, increases BiP levels, without altering Chop or Xbp1s levels (Pennuto et al., 2008). This may be another example where UPR mediators play roles in homeostasis during development. For example, Xbp1s is required for normal differentiation of plasma cells, which like Schwann cells, also express immunoglobulin family members (Reimold et al., 2001; Iwakoshi et al., 2003; Rutkowski and Hegde, 2010).

Repeated attempts to measure protein translation rates in S63del and S63del//*Perk*+/− nerves were performed as previously described (D’Antonio et al., 2013), but the method was not sensitive enough to reveal a difference between these two genotypes (not shown). Nonetheless, the 40% decrease of P-eIF2alpha levels was sufficient to promote alterations in myelination in S63del nerves. Similarly, partial ablation of *Perk* was sufficient to produce associated phenotypic changes in mouse models of central demyelination (W. Lin et al., 2005, 2006).

### PERK is Detrimental for S63del Myelination

PERK through phosphorylation of eIF2alpha, reduces protein translation rates for almost 90% of cellular mRNAs (Harding et al., 2001a; Ventoso et al., 2012). When we attenuated translation by interfering with Gadd34, and increasing eIF2alpha phosphorylation, we were able to relieve the levels of UPR activation and achieve almost complete morphological and functional rescue of S63del neuropathy (D’Antonio et al., 2013). Therefore, we performed the converse experiment in *Perk*+/− or S63del//*Perk*+/− mice. Perk ablation in mice without the P0S63del transgene reduced eIF2alpha phosphorylation, and had a detrimental effect on motor performance. Moreover, myelination in *Perk*+/− and *Perk*−/− DRG explants was impaired and the length of internodes was shorter than WT (Figure 4b–d). These data suggest that Perk is beneficial for myelination in WT.

In contrast, and to our great surprise, Perk deficiency in S63del mice did not worsen, but ameliorated morphological and functional features of neuropathy (Figures 2, 3, and 5). Onion bulbs and demyelinated fibers, the hallmarks of S63del neuropathy, were rescued in S63del//*Perk*+/− animals (Figure 3). Functionally, motor performance was significantly ameliorated in S63del//*Perk*+/− animals, strongly suggesting that *Perk* contributes a detrimental effect to the pathogenesis of S63del neuropathy (Figure 2).

In *Perk*+/− mice, *Perk* ablation may negatively influence motor phenotype not only by acting in peripheral nerves, but also in other tissues. In this mouse model, one *Perk* allele is ablated in all cells of the body, and *Perk* haploinsufficiency is known to have negative consequences in several organs (Harding et al., 2001b)—some crucial for motor function (i.e., brain, spinal cord, or muscle; Jones and Roberts, 1968; Hamm et al., 1994; Rustay et al., 2003; Minasyan et al., 2009; Shiotsuki et al., 2010). For example, in the brain, it is possible that *Perk* haploinsufficiency can interfere with the motor learning phase of rotarod analysis or myelination (W. Lin and Popko, 2009; Trinh et al., 2012), whereas, in muscle, *Perk* ablation can impair myocyte Ca^2+^ signaling and contraction (Huang et al., 2006). Another possibility is that *Perk* ablation could contribute to S63del neuropathy in a non-nerve autonomous way, but without obvious morphological changes outside of nerve. For example, a systemic metabolic disturbance like glucose intolerance (Harding et al., 2001b) could secondarily affect neurons or Schwann cells negatively. Thus, nerve-independent effects could contribute to the poor rotarod performance of *Perk*+/− mice, even if morphology and NCV studies of sciatic nerve appear grossly normal (Figure 2).

However, these same detrimental effects are not likely to explain the altered rotarod performance in S63del//*Perk*+/− mice, as it improved relative to S63del. Accordingly, we did not document any correlating morphological alteration in cerebellum, spinal cord, or muscle, apart from a very slight increase (from 0.5% to 1.5%) of centrally nucleated fibers in S63del gastrocnemius muscle, that returned toward normal in S63del//*Perk*+/−. Centrally nucleated fibers are linked with muscle degeneration/regeneration. However, 1–2% centrally nucleated fibers have been reported in normal young adult mouse muscle (Sonnemann et al., 2006). In contrast, clinically symptomatic mice with early muscular dystrophy typically have already 30–60% centrally nucleated fibers (Nguyen et al., 2002).

### Perk-Null Neurons Do Not Alter Myelination by S63del Schwann Cells

To eliminate a possible role for other tissues in the improvement of S63del//*Perk*+/− mice, we studied the effect of Perk ablation on myelination in cultures containing only Schwann cells and neurons. We isolated DRGs from S63del//*Perk*−/− embryos and induced myelination *in vitro* (Taveggia et al., 2005). This experiment also allowed us to study complete ablation of *Perk*. In S63del//*Perk*+/− and S63del//*Perk*−/− myelinating explant cultures, the increased internodal length nicely correlated with the rescue observed in S63del//*Perk*+/− sciatic nerves, suggesting, once more, that *Perk* has a detrimental function for S63del myelination (Figure 4). Myelination depends on carefully orchestrated, reciprocal signals between neurons and Schwann cells (Taveggia et al., 2010). We then asked whether altering *Perk* and eIF2-alpha phosphorylation in neurons could influence myelination, in a non-cell autonomous manner. To test this hypothesis, we cultured *Perk*-deficient neurons with WT rat Schwann cells, already known to myelinate mouse neurons normally (Taveggia et al., 2005). The high rate of myelination observed in all genotypes analyzed indicates that *Perk*-deleted neurons do not alter myelination. These data, together with the results of DRG explant myelination, suggest that *Perk* influences myelination in a Schwann cell autonomous manner. Future experiments may address this *in vivo*, by deleting *Perk* in Schwann cells and neurons using conditional ablation of *Perk* with Cre-recombinases (P. Zhang et al., 2002a).

### S63del//*Perk*+/− Rescue is not correlated with Alterations in Chop and Gadd34

It has been shown that Chop and Gadd34 are detrimental in S63del neuropathy. In fact, the complete ablation of Chop or Gadd34 rescued S63del neuropathy *in vivo* (Pennuto et al., 2008; D’Antonio et al., 2013). Although they are downstream of PERK, surprisingly, Chop and Gadd34 mRNA expression were not reduced in S63del//*Perk*+/− compared with S63del sciatic nerves. This is consistent with previous reports showing that *Chop* can be upregulated independently by ATF6 (Yoshida et al., 2000). Additionally, CHOP remains detected in nuclei of myelinating Schwann cells, and CHOP target genes remain activated, suggesting that CHOP loss of function does not explain rescue in S63del//*Perk*+/− mice.

Gadd34 protein levels, instead, drop by about half in absence of PERK (Figure 6e). This can be explained by the post-transcriptional regulation of Gadd34 mRNA mediated by the uORF regions in the 5′ UTR of its mRNA, similar to ATF4 (Harding et al., 2000a). Gadd34/PP1 holoenzyme promotes eIF2alpha dephosphorylation and restarts protein translation after initial pausing (Brush et al., 2003; Marciniak et al., 2004). Complete genetic ablation of Gadd34 rescues S63del neuropathy phenotype by impeding reversal of protein synthesis (D’Antonio et al., 2013); haploinsufficiency of Gadd34 had no effect on S63del neuropathy. Even if S63del//*Perk*+/− mice resemble S63del//*Perk*+/−-deficient mice functionally and morphologically, the amelioration observed in this study is not correlated with eIF2alpha phosphorylation levels. In S63del//*Perk*+/− mice, eIF2alpha is hypo-phosphorylated (Figure 1d; 40% lower than S63del mice), whereas in S63del/*Gadd34*-deficient mice, eIF2alpha is overphosphorylated (30% higher; D’Antonio et al., 2013). This indicates that translational homeostasis is not the main mechanism of rescue in S63del//*Perk*+/− mice.

The role of the *Perk* pathway in neurodegenerative and other ER stress-related diseases is still controversial. For instance, PERK is beneficial in models of central nervous system (CNS) and PNS demyelination (W. Lin et al., 2006; D’Antonio et al., 2009; D’Antonio et al., 2013; W. Lin et al., 2013; Y. Lin et al., 2014). In contrast, *Perk* elimination is protective in prion disease and in the diabetic Akita mice (Gupta et al., 2010; Moreno et al., 2012; Moreno et al., 2013).

A possible explanation is that Perk ablation elicits alternative and beneficial responses to cope with stress in a context-dependent manner. For example, *Perk*-null cells challenged with tunicamycin, an ER stress inducer, manifest prolonged IRE1 activation (Harding et al., 2000b; A. H. Lee et al., 2002; K. Lee et al., 2003; Walter and Ron, 2011). Therefore, we reasoned that the lack of PERK in S63del could induce other UPR pathways to compensate. However, our data show that Perk haploinsufficiency did not alter either the IRE1 or ATF6 pathways in S63del nerves.

Collectively, our data show that *Perk* ablation paradoxically improves S63del neuropathy despite that eIF2alpha phosphorylation is reduced in nerve. We thus conclude that the translational homeostasis in Schwann cells is not the only factor involved in the pathogenesis of CMT1B. For example, we cannot rule out a possible effect of beneficial effect of *Perk* deletion in neurons or other cells, which may contribute to the phenotype observed in S63del//*Perk*+/− mice. In order to study the cell autonomous effect of *Perk,* we will need to delete *Perk* specifically in neurons or Schwann cells in the S63del mice in a future study.

## Summary Statement

PERK kinase senses stress in the ER and attenuates translation through eIF2alpha phosphorylation, a beneficial response in many ER stress-related diseases. Nonetheless, the authors report that *Perk* ablation surprisingly improves CMT1B peripheral neuropathy.

## Supplementary Material

Supplementary material
