# Supplementary material for: Perk Ablation Ameliorates Myelination in S63del-Charcot–Marie–Tooth 1B Neuropathy
Source: ASN Neuro. 2016 Apr 14;8(2):1759091416642351. doi: 10.1177/1759091416642351 (PMC4844932; doi:10.1177/1759091416642351)
Supplement: Supplementary material [file Supplementary_Figures.pdf]

SUPPLEMENTARY MATERIAL:
Perk ablation ameliorates myelination in S63del-Charcot-Marie-Tooth 1B neuropathy

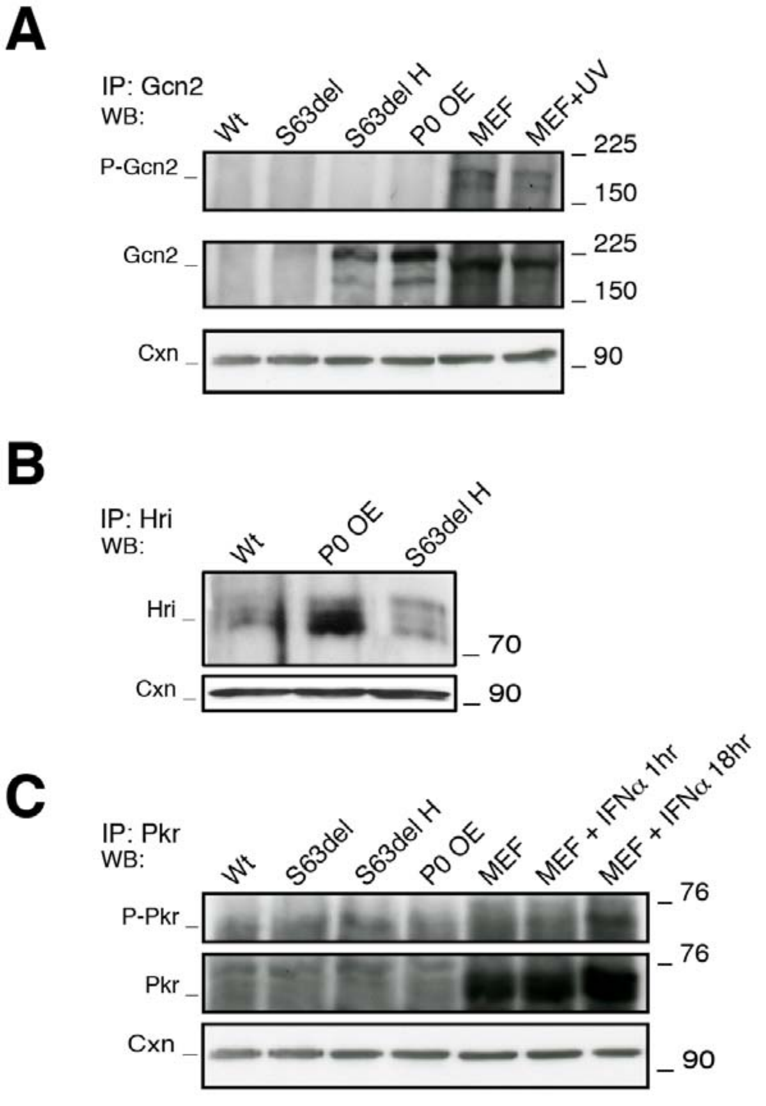

Supplementary Figure 1. GCN2, HRI and PKR are not activated in S63del sciatic nerves.

A-C: Immunoprecipitation (IP) followed by Western blot (WB) for GCN2, or P-GCN2 (Thr898); for HRI; and for PKR or P-PKR (Thr446) on P28 sciatic nerve extracts. S63del low (S63del) and high (S63del H) express the P0S63del transgene at different levels (60% and 210% overexpression, respectively). Negative control lysates from P0OE sciatic nerve and positive control lysates from mouse embryonic fibroblast (MEF) treated with ultraviolet light (+UV) or interferon-α (+IFNα) were analyzed. Numbers represent relative molecular weight standards. Calnexin (Cxn) was used as a loading control. One representative blot of 2 replicates is shown.

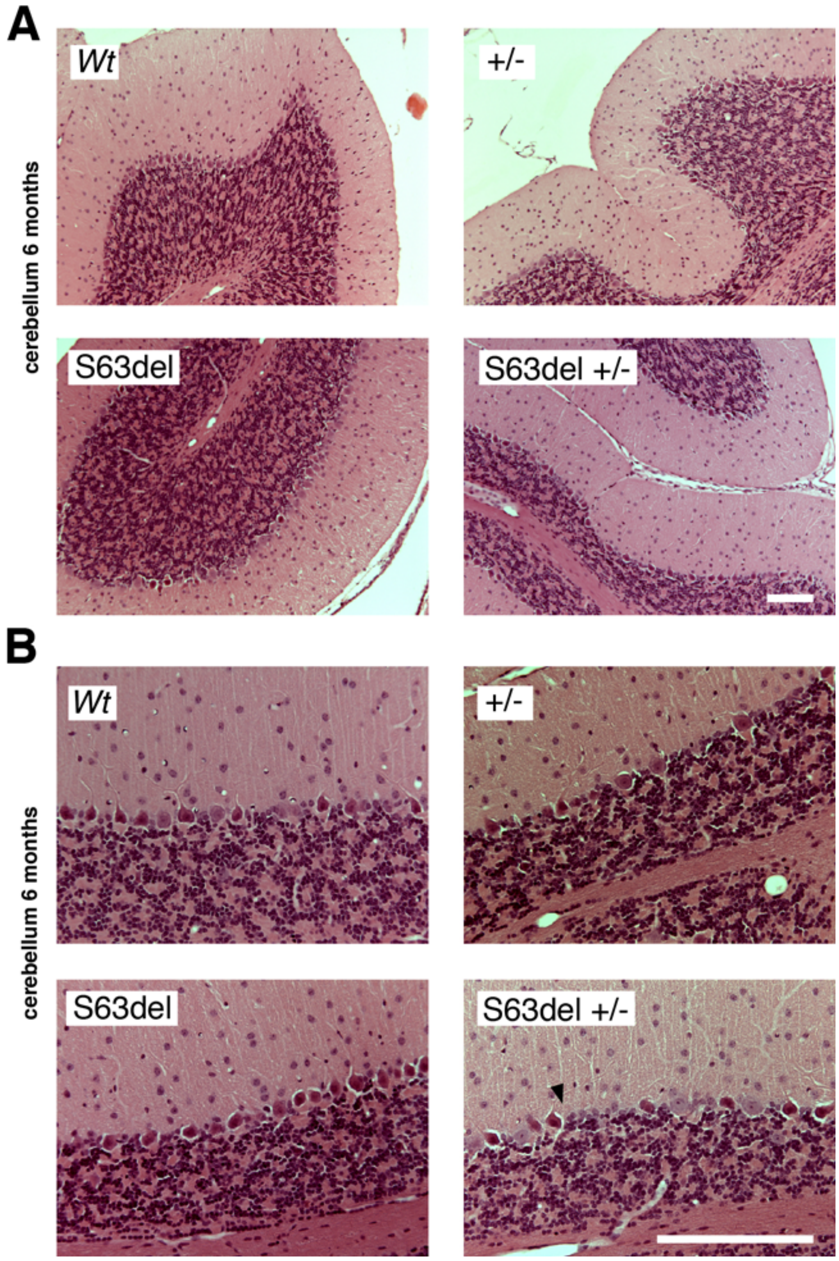

Supplementary Figure 2. Morphology of the cerebellum is not affected by Perk haploinsufficiency.

A: Hematoxilin/eosin staining of cerebellar sections from 6 month old WT, *Perk+/-*, S63del and S63del/*Perk+/-* mice. **B**: 3.3-fold magnification of sections in **A**. A representative section of three animals per genotype is shown. Occasional discontinuity of the Purkinje cell layer was noted in S63del/*Perk+/-* (arrowhead), but the number of Purkinje cells was not altered. Size bar, 100μm.

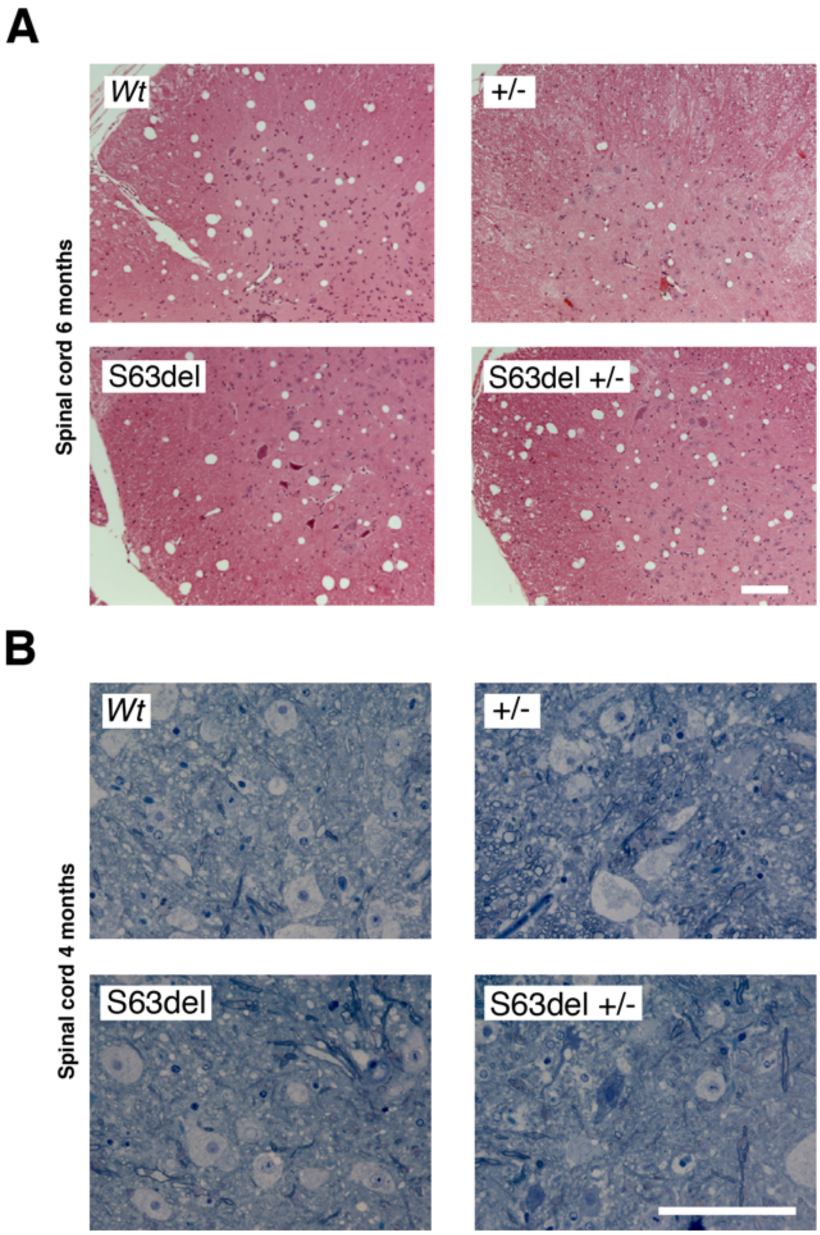

Supplementary Figure 3. Morphology of spinal cord ventral horns is not affected by Perk haploinsufficiency.

A: Hematoxilin/eosin staining of spinal cord sections from 6 month old WT, *Perk+/-*, S63del and S63del/*Perk+/-* mice. **B:** Toluidine blue staining of spinal cord sections (ventral horns) from 4 month-old mice. A representative section of three animals per genotype is shown. Size bar, 100μm.

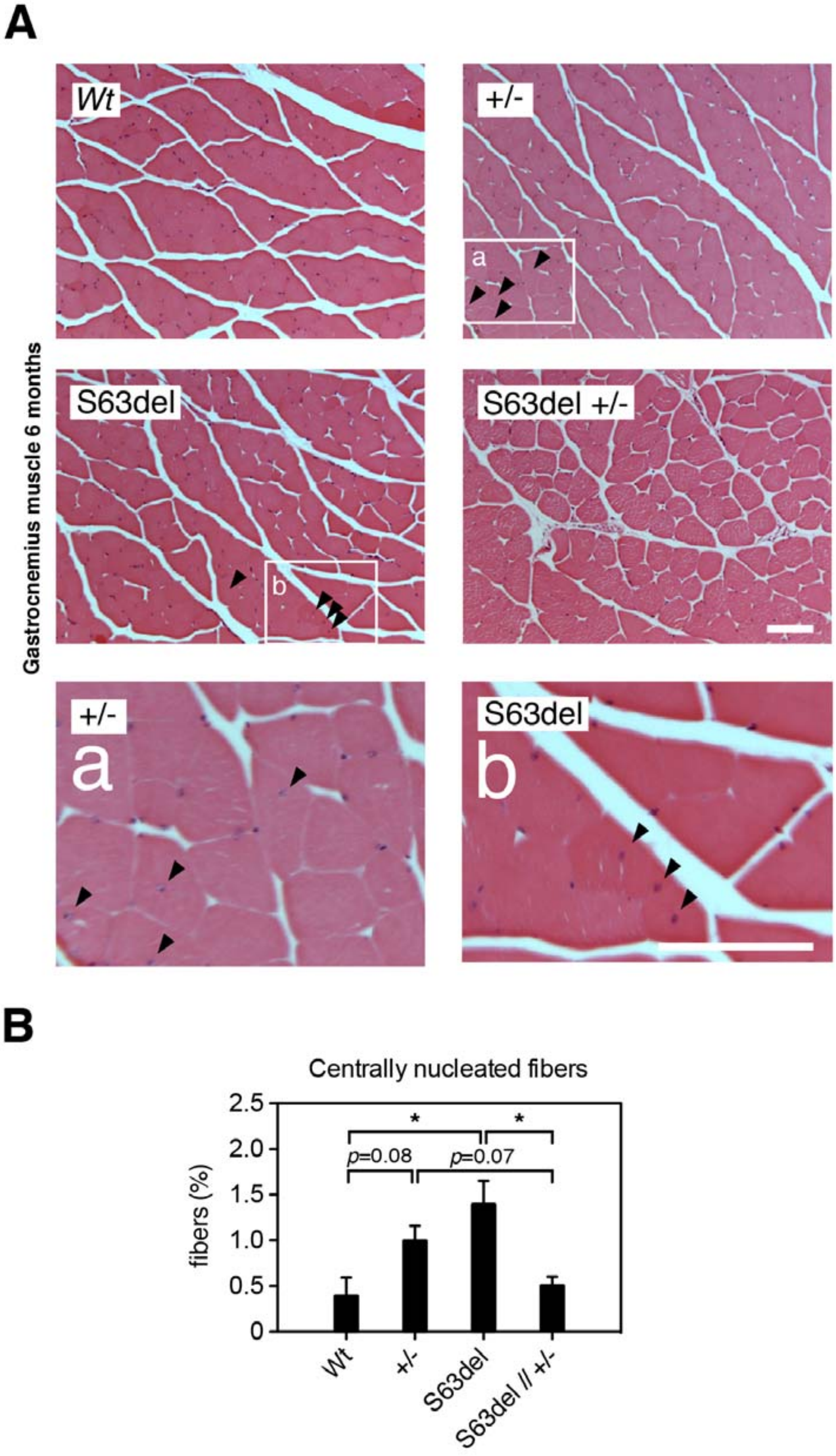

Supplementary Figure 4. Gastrocnemius muscles display a PERK-dependent increase in centrally nucleated fibers in S63del muscle.

A: Hematoxilin/eosin staining of muscle sections from 6 month-old WT, *Perk+/-*, S63del and S63del/*Perk+/-* mice. Black arrowheads indicate centrally nucleated muscle fibers (in a and b, 3.5-fold magnifications of insets are shown). A representative section obtained from three animals per genotype is shown. Size bar, 100μm. **B:** Proportion of centrally nucleated fibers in muscle sections. Error bars, SEM; *, $p<0.05$; by Student's *t*-test; $n=3$.

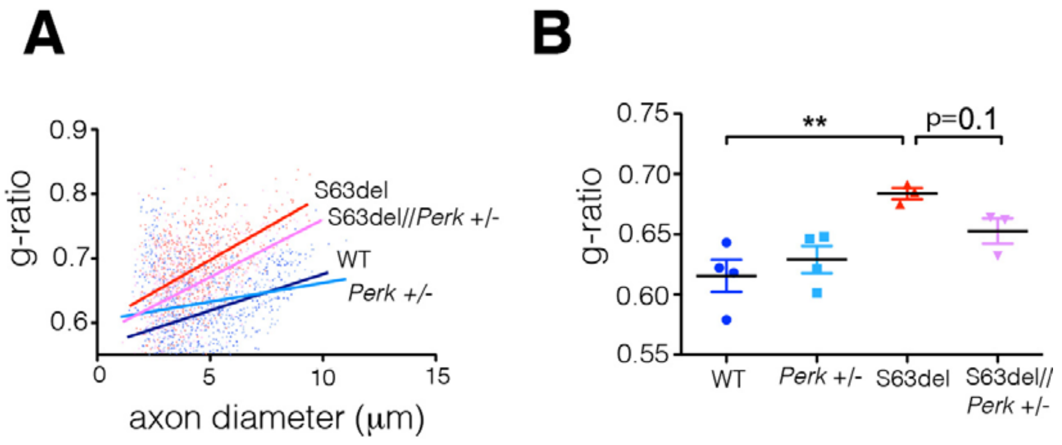

Supplementary Figure 5. Myelin thickness in S63del and S63del//*Perk*+/- nerves at 12 months.

A: Scatter plot of g-ratios as a function of axonal diameter are shown for WT, *Perk*+/-, S63del and S63del/*Perk*+/- mice at 12 months old (*n*=3-4). **B:** A graphic representation is shown of the average g-ratio for each genotype. Error bars, SEM; **, $p<0.01$, by One-way Anova with Bonferroni comparison; *15-20* fields derived from *n*=3-4 animals per genotype

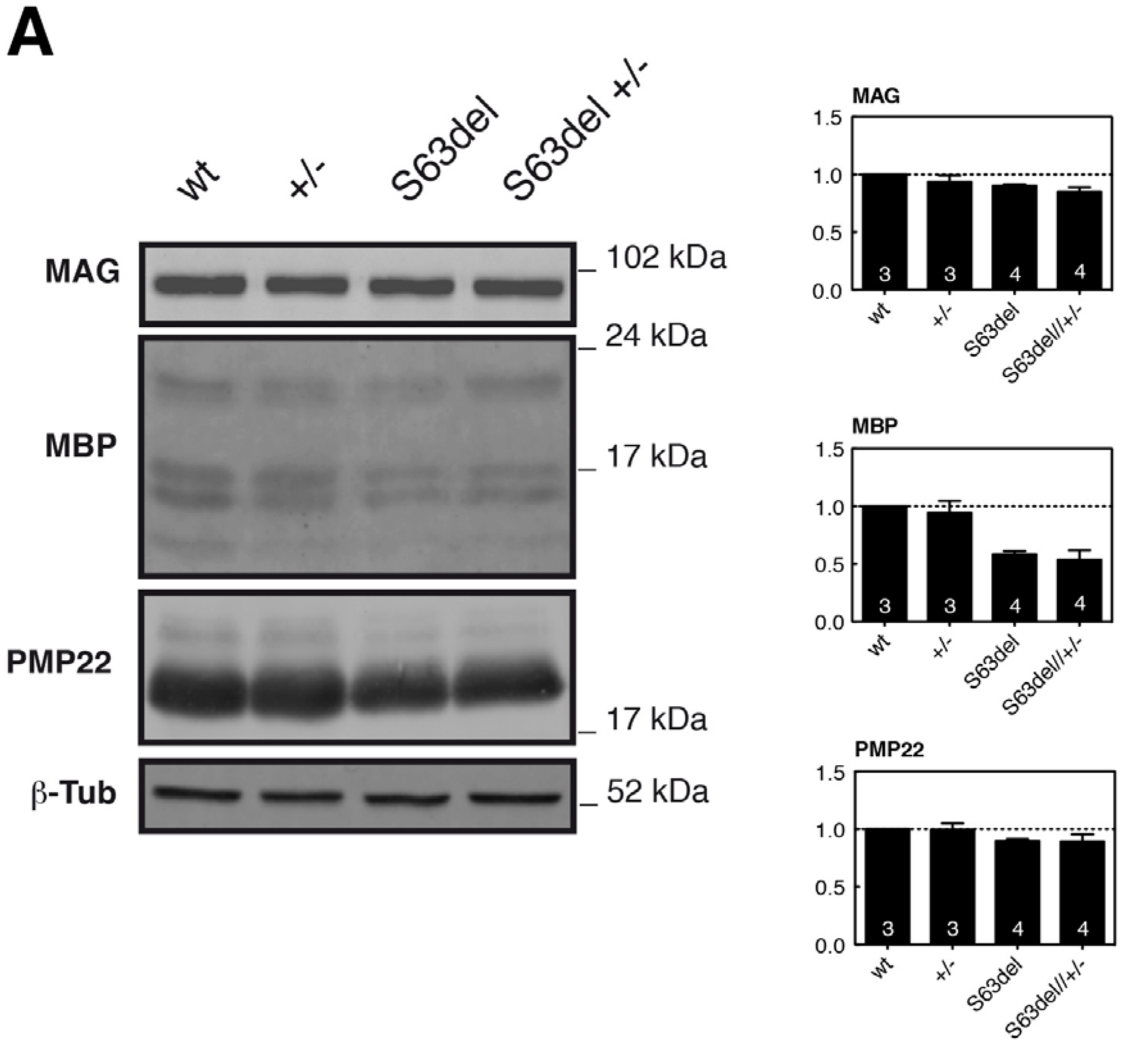

Supplementary Figure 6. ***Perk*** **haploinsufficiency does not alter the levels of myelin proteins in WT or S63del sciatic nerves at P28.**

A: *Perk*+/- and WT myelin protein levels are comparable as measured by western analysis. MBP levels are reduced in S63del as compared to WT. However, the levels of MAG, MBP and PMP22 are very similar in S63del/Perk+/- versus S63del. β-Tubulin was used as loading control. The graphs represent the mean±SEM relative to WT set as 1.
